# Case report: Adult-onset limb girdle muscular dystrophy in sibling pair due to novel homozygous *LAMA2* missense variant

**DOI:** 10.3389/fneur.2023.1055639

**Published:** 2023-01-27

**Authors:** Matthew Katz, Leigh B. Waddell, Michaela Yuen, Samantha J. Bryen, Emily Oates, Fleur C. Garton, Thomas Robertson, Robert David Henderson, Sandra T. Cooper, Pamela A. McCombe

**Affiliations:** ^1^Department of Neurology, Royal Brisbane and Women's Hospital, Herston, QLD, Australia; ^2^Kids Research, Kids Neuroscience Centre, The Children's Hospital at Westmead, Sydney, NSW, Australia; ^3^Discipline of Child and Adolescent Health, Faculty of Medicine and Health, The University of Sydney, Sydney, NSW, Australia; ^4^School of Biotechnology and Biomolecular Sciences, University of New South Wales, Sydney, NSW, Australia; ^5^Institute for Molecular Biosciences, The University of Queensland, St Lucia, QLD, Australia; ^6^Department of Pathology, Royal Brisbane and Women's Hospital, Herston, QLD, Australia; ^7^School of Biomedical Sciences, The University of Queensland, St Lucia, QLD, Australia; ^8^The Children's Medical Research Institute, Sydney, NSW, Australia; ^9^Centre for Clinical Research, The University of Queensland, Herston, QLD, Australia

**Keywords:** limb girdle muscular dystrophy, genotype, phenotype, *LAMA2*, whole exome sequencing

## Abstract

Recessive pathogenic variants in the laminin subunit alpha 2 (*LAMA2*) gene cause a spectrum of disease ranging from severe congenital muscular dystrophy to later-onset limb girdle muscular dystrophy (LGMDR23). The phenotype of LGMDR23 is characterized by slowly progressive proximal limb weakness, contractures, raised creatine kinase, and sometimes distinctive cerebral white matter changes and/or epilepsy. We present two siblings, born to consanguineous parents, who developed adult-onset LGMDR23 associated with typical cerebral white matter changes and who both later developed dementia. The male proband also had epilepsy and upper motor neuron signs when he presented at age 72. Merosin immunohistochemistry and Western blot on muscle biopsies taken from both subjects was normal. Whole exome sequencing revealed a previously unreported homozygous missense variant in *LAMA2* [Chr6(GRCh38):g.129297734G>A; NM_000426.3:c.2906G>A; p.(Cys969Tyr)] in the proband. The same homozygous *LAMA2* variant was confirmed by Sanger sequencing in the proband's affected sister. These findings expand the genotypic and phenotypic spectrum of LGMDR23.

## Introduction

Laminin subunit alpha 2 (*LAMA2*) encodes the α2 subunit of merosin (laminin-2), a glycoprotein critical for linking the sarcolemma to the extracellular matrix (1). Recessive pathogenic *LAMA2* variants cause a wide spectrum of dystrophic muscle disease ranging from severe congenital muscular dystrophy to later-onset limb girdle muscular dystrophy (LGMDR23), which presents after 12 months of age (2). LGDMR23 is a rare form of LGMD, accounting for only about 2% of all patients in Western European LGMD cohorts (3, 4). Most cases of LGMDR23 are caused by missense variants that result in partial merosin deficiency (2).

LGMDR23 typically presents in childhood with slowly progressive proximal limb weakness, contractures and raised creatine kinase (CK) (2). Motor milestones can be delayed, though nearly all patients achieve independent ambulation, which is typically maintained throughout life (2). Central nervous system (CNS) complications feature prominently in LGMDR23, with distinctive cerebral white matter signal changes reported in almost all cases and epilepsy in some (2).

Here we describe a proband aged 72 years who presented in his early thirties with LGMD and progressed to develop severe dementia, which began manifesting in his mid-sixties, and diffuse upper motor neuron (UMN) signs without a clear alternative explanation for these features. His sister also had a history of LGMD followed by the development of dementia in her late fifties. Whole exome sequencing (WES) in the proband identified a previously unreported homozygous missense variant in *LAMA2* [Chr6(GRCh38):g. 129297734G>A; NM_000426.3:c.2906G>A; p.(Cys969Tyr)]. The same homozygous *LAMA2* variant was confirmed by Sanger sequencing in the proband's affected sister. Although cerebral white matter changes are common, UMN findings and severe cognitive impairment have not previously been reported in association with LGMDR23.

## Methods

### Ethics

Ethical approval for use of muscle tissue and DNA from probands and controls were obtained from the Human Research Ethics Committees of the Children's Hospital at Westmead, Australia (2019/ETH11736) and all study participants (or their guardians) provided informed, written consent.

### Genetic testing

Whole exome sequencing was performed for the proband as part of a previous study, where the proband remained undiagnosed (5). The WES data was reanalysed in the present study. Variant filtering and analysis was performed in seqr (6) using a minor allele frequency cut-off of 0.001 from the gnomAD database (7) and genes associated with neuromuscular disorders in publicly available gene lists (8) (Supplementary Table 1). Sanger sequencing was performed to confirm the *LAMA2* variant in the proband and his affected sister, using the primers; LAMA2ex21F: 5'-agttcctctgttcccacctg-3' and LAMA2ex21R: 5'-cgttgtatcaatctgtgcttcc-3.'The chromosomal microarray (CMA) was performed using the Illumina Whole-Genome Infinium CytoSNP 850K Array v1.1. Results were analyzed with BlueFuse Multiversion 4.4, using genome reference sequence GRCh37/hg19.

### Muscle immunohistochemistry and western blot

Cryopreserved muscle tissue was sectioned at 8 μm thickness and collected in pre-cooled microcentrifuge tubes for western blot or on microscopic slides (SuperFrost^®^Plus, Menzel, Germany) for immunostaining.

Immunostaining was performed on non-fixed tissue as described previously (9) using α-merosin (laminin α2) antibodies (MAb1922, Chemicon, 1:20000; NCL-Merosin, Novocastra, 1:200) and α-spectrin antibody (NCL-SPEC1, Novocastra, 1:300). Slides were imaged on a Leica DMi8 microscope and figures prepared using Adobe Illustrator.

Protein sample preparation and western blot was performed as previously described (10). Protein samples were separated on a 4–12% 1 mm NuPAGE™ Bis-Tris gel (Life Technologies) using MOPS running buffer at 150 V and transferred onto a PVDF membrane (Merck Millipore Immobilon™-P, 0.45 μm) for 1 h at 400 mA in Tris-Glycine buffer containing 0.075% sodium dodecyl sulfate and 15% methanol. Total protein was detected using Revert™ 700 total protein stain (LI-COR Biosciences) according to manufacturer's instructions.

Membranes were cut at the 115kD marker band and probed with an α-merosin antibody (bottom half, MAB1922, Chemicon, 1:500) and α-dystrophin antibody (top half, NCL-DYS3, Novocastra, 1:100) overnight at 4°C. Antibody binding was detected using an α-mouse HRP secondary antibody (Merck, 1:1000 for DYS3 detection and 1:2000 for merosin detection) for 2 h at room-temperature. Blots were imaged using the Odyssey CLx imaging system (LI-COR Biosciences) and analyzed using ImageJ. Merosin protein levels were normalized to actin (from total protein stain) and dystrophin. Dystrophin protein levels were also normalized to total protein. The average of 2–3 experiments for each sample was plotted on the graph.

## Case report

**Figure 1:**
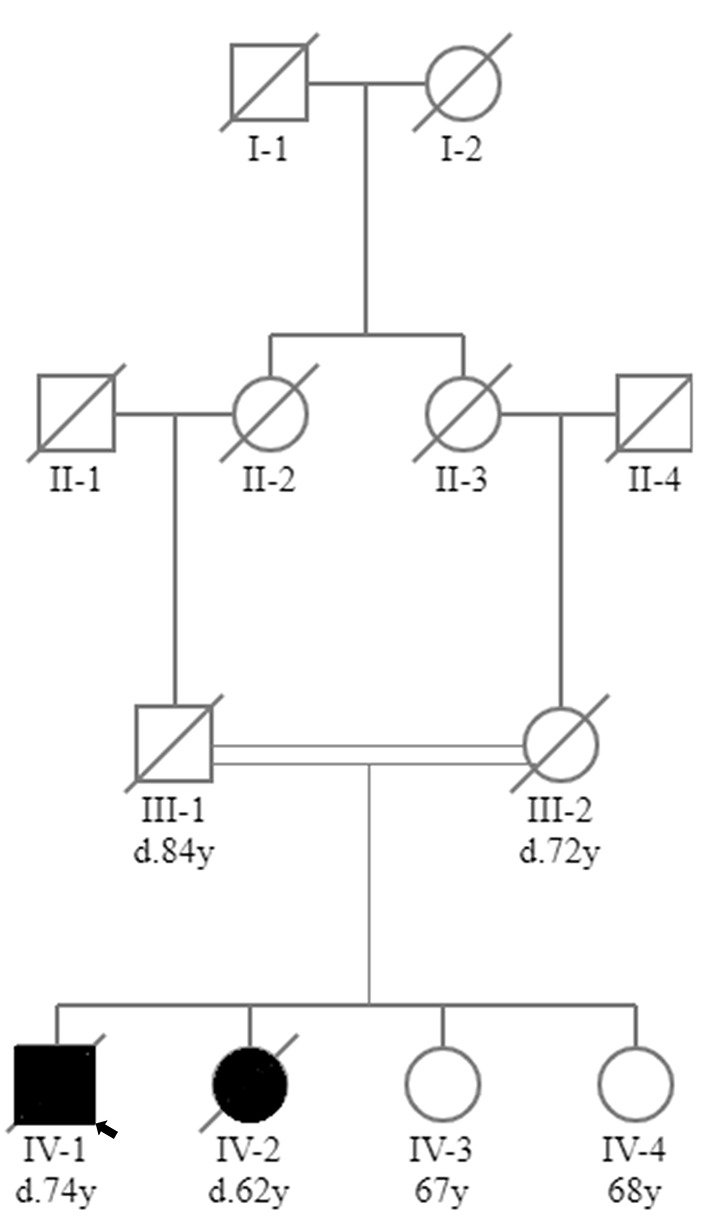
Family tree. Family tree showing that the proband (IV-1) and his affected sister (IV-2) were born to consanguineous first cousin parents (III-1 and III-2). Two sisters in their 60s were unaffected (IV-3 and IV-4). Square, male; circle, female; black circle or square, clinically affected; white circle or square, clinically unaffected; line through circle or square, deceased (age of death, where known, given below circle or square as denoted by d.); double line, consanguinity; small black arrow, proband.

**Figure 2:**
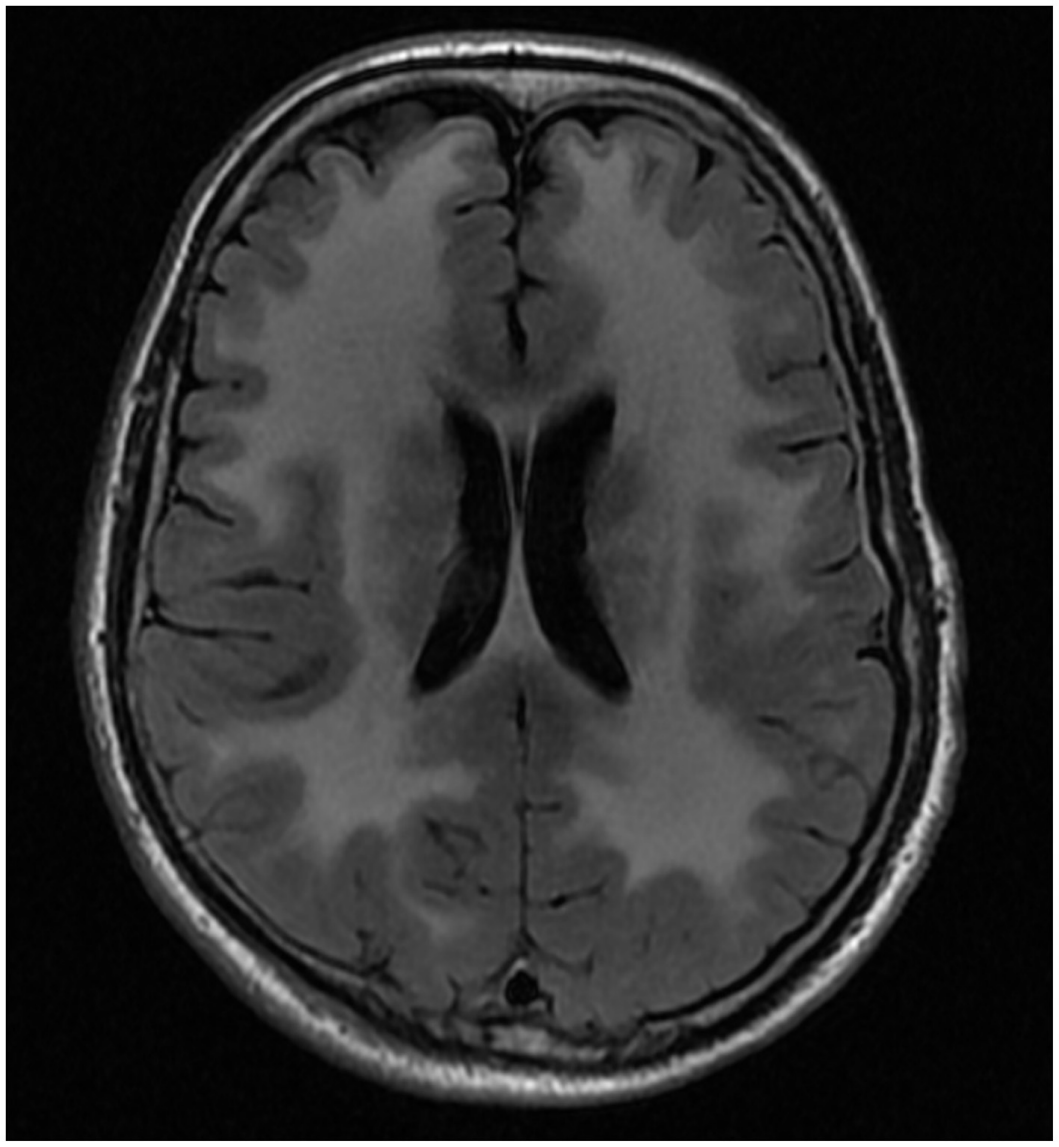
MRI brain of proband. Axial FLAIR^*^ image from MRI brain of proband at 64 years of age showing confluent, bilateral white matter T2# hyper intensity with involvement of the corpus callosum ^*^FLAIR, Fluid-attenuated inversion recovery; #T2, transverse relaxation time.

The male proband (IV-1) was the first child of consanguineous first cousin parents (III-1 and III-2) (Figure 1). He had three sisters (IV-2, IV-3 and IV-4). He had a history of normal childhood motor and language development and was University educated. He presented at age 36 years with a several year history of frequent falls, initially attributed to pelvic fractures sustained from a previous mining accident. He experienced slowly progressive limb weakness with elevated CK (400–600 U/L; normal range <171 U/L). Cognitive issues manifested in his mid-60 s, with family members reporting poor decision making, reduced insight and memory problems. There were no visual hallucinations. Progressive cognitive impairment and severe weakness necessitated high-level nursing home care. Tonic-clonic epileptic seizures, without clear focal onset, began in his late 60 s. Magnetic resonance imaging (MRI) of the brain aged 64 showed confluent, bilateral white matter T2 hyperintensity with involvement of the corpus callosum (Figure 2). There was no significant cerebral or hippocampal atrophy. At around 70 years of age the proband became dependent upon the use of a wheelchair, non-verbal and incontinent.

The proband had a history of well-controlled type II diabetes mellitus, hypothyroidism, depression, paroxysmal atrial fibrillation, and recurrent squamous cell carcinomas. His regular medications at age 72 included sodium valproate 600 mg twice daily, aspirin 100 mg daily, thyroxine 50 mcg 5/7 days and 100 mcg 2/7 days, metformin 500 mg daily, vitamin D 25 mcg daily, insulin glargine 25 units subcutaneously daily, Movicol sachet daily, docusate sodium with senna two tablets twice a day, paracetamol 500 mg two tablets four times a day and risperidone 2 mg as needed.

Examination at the age of 72 showed that he could only follow basic one-step commands and was non-verbal precluding more formal cognitive testing. There was diffuse limb muscle wasting with fixed flexion contractures of the elbows, fingers, hips, and knees. There was Medical Research Council (MRC) grade ≤3 weakness of the proximal and distal limb muscles that could be tested. There was calf pseudohypertrophy. Limb tone was difficult to assess due to the presence of contractures, but pathologic clonus was elicited at the ankles. The reflexes were brisk, and the plantar responses were equivocal. Sensory examination was challenging but the patient appeared to feel sharp sensation in all four limbs. His pupils were equal and reactive to light. Detailed examination of eye movements was not possible due to his difficulty in following instructions and sustaining attention. No facial weakness was observed. The proband was unable to stand or walk.

There was no record of a previous electroencephalogram, nerve conduction study/electromyogram (EMG), transthoracic echocardiogram or respiratory function test. Given the degree of his disability and advanced dementia these were not pursued as the results would not have changed management. The proband continued to decline and poor control of his tonic-clonic seizures resulted in an escalation of his anti-seizure medications. The proband succumbed to aspiration pneumonia at the age of 74.

His younger sister (IV-2) died at the age of 62 from pneumonia in the setting of early onset dementia. She presented to another facility with slowly progressive limb weakness with elevated CK (400-650 U/L) and gait dysfunction from the age of 50. Poor decision making and impairments in working memory were noted in her late 50 s. A limited EMG of her biceps muscle showed myopathic changes characterized by small amplitude motor units without abnormal spontaneous activity. Neuroimaging showed extensive cerebral white matter disease (not shown). From the age of 59 there was a more rapid deterioration in her cognitive and physical function culminating in her being bedbound, mute, and doubly incontinent for the 12 months prior to her death. She did not have a history of epilepsy. The other two sisters in their 60 s (IV-3 and IV-4) were unaffected. There was no other family history of neuromuscular disease or dementia.

### Genetic testing

Genetic testing, including targeted sequencing of *FKRP, DNM2* and *LARGE* genes, failed to identify causative genetic variants. Whole exome sequencing, performed from blood of proband (IV-1) identified a previously unreported homozygous missense variant [NM_000426.3:c.2906G>A;p.(Cys969Tyr)] in exon 21 of *LAMA2* (Table 1) [submitted to ClinVar–https://www.ncbi.nlm.nih.gov/clinvar/variation/1693116/?oq=LAMA2%5bgene%5+p.Cys969Tyr&m=NM_000426.4(LAMA2):c.2906G>A%20(p.Cys969Tyr)]. Sanger sequencing confirmed the *LAMA2* variant was homozygous in the proband and his affected sister (IV-2). The two unaffected sisters (IV-3 and IV-4) were not available for genetic testing for segregation analysis. A chromosomal microarray detected multiple long contiguous stretches of homozyosity (LCSH), including the *LAMA2* locus on chromosome 6.

**Table 1:** Genetic summary table for novel variant.

**Gene**	**Transcript reference**	**hg38**	**Codon variant**	**Amino acid change**	**POPmax**	*In silico* prediction*	**ACMG-AMP classification**
*LAMA2*	NM_000426.3	6:129297734	c.2906G>A	p.Cys969Tyr	NR	DDD	Likely pathogenic (PM2, PM3^#^, PP1, PP3)

hg38, human reference genome; POPmax, maximum allele frequency in each reference population from 1000G, ExAC, gnomAD and ESP; ACMG-AMP, American College of Medical Genetics and Genomics - Association for Molecular Pathology (version: 8.4.7 from Varsome); NR, allele not previously reported.

* Meta SVM, Meta LR and M-Cap in silico prediction (“D” = deleterious).

# assumed parental carriers due to consanguinity (first cousins), confirmed in case with LCSH via chromosomal microarray.

### Muscle histopathology

**Figure 3:**
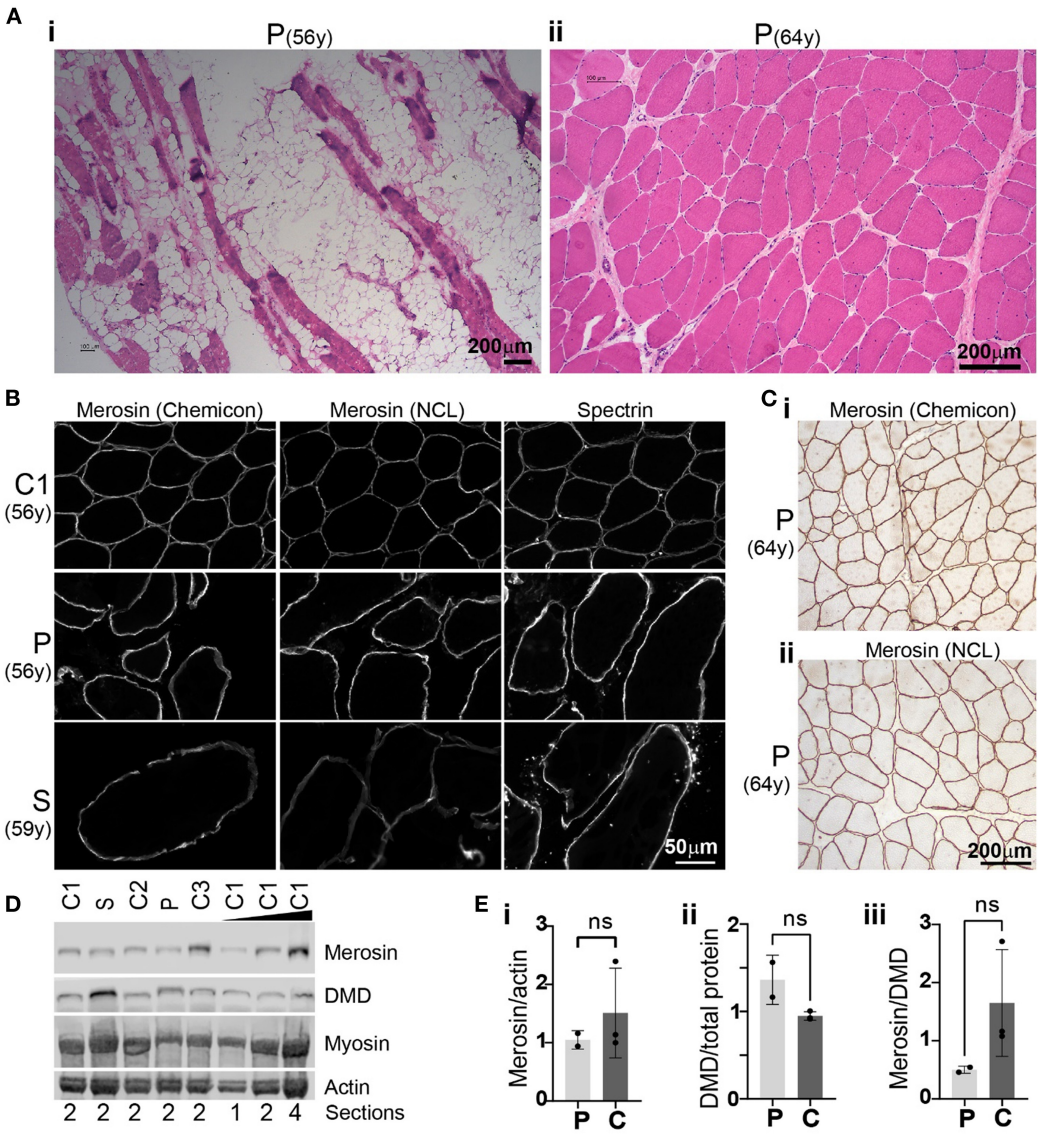
Histology, immunohistochemistry and Western blot analysis. **(A)** Haematoxylin and eosin (H&E) staining of muscle biopsies obtained from the proband (P) at age 56 years (i; quadriceps) and 64 years (ii; biceps). Unstained areas observed in (i) represent adipose tissue infiltrates while muscle fibers appear pink in H&E-stained sections. **(B)** Merosin immunohistochemical staining of proband (P) quadriceps muscle biopsies and a muscle biopsy obtained from the proband's sister (S) at 59 years demonstrating membrane-staining comparable to control 1 (C1). Immunostaining for spectrin, another membrane-associated protein, showed a staining pattern similar to merosin. **(C)** Immunohistochemical staining of proband biceps muscle obtained at age 64 years with two merosin antibodies (i: Chemicon MAb1922; ii: NCL-merosin Novocastra) demonstrated membrane-associated staining pattern of merosin similar to **(B)**. **(D)** Western blot of muscle from the proband (P; 56y) and his sister (S; 59y) showed merosin protein levels similar to those detected in three control samples: C1 = 56y (female), C2 = 57y (female), C3 = 59y (male). **(E)** Bar graphs of merosin protein levels normalized to actin (measured from total protein stain) (i), dystrophin protein levels normalized to total protein (ii) and merosin protein levels normalized to dystrophin (iii). Average ± standard deviation for proband and sister (collectively denoted as P) and controls (C) are shown. Groups were compared by using a t-test and found to be not significantly different (ns, i; p = 0.4848, ii; 0.0734, iii; 0.1926).

A quadriceps muscle biopsy obtained from the proband at age 56 years showed myopathic changes with marked replacement of muscle with adipose tissue (Figure 3Ai). The remaining muscle fibers showed marked variation in myofibre diameter with numerous severely atrophic fibers.

A biceps muscle biopsy taken subsequently, aged 64 years, showed less severe myopathic changes compared to his quadriceps biopsy with increased internal nuclei, variation in myofibre diameters including hypertrophic and atrophic fibers, but no adipose tissue infiltration (Figure 3Aii). A quadriceps muscle biopsy of the proband's sister at age 59 years showed myopathic changes similar to the quadriceps muscle biopsy from her brother at the age of 56 with severe variation in myofibre diameter, numerous atrophic fibers and significant replacement of skeletal muscle with fibroadipose tissue (not shown).

To determine whether the homozygous missense variant in *LAMA2* results in abnormal subcellular localization and abnormal protein levels of the encoded protein merosin, we performed immunohistochemistry and western blot analysis. Merosin immunohistochemistry on two biopsies from the proband (age 56 and 64 years) and one biopsy from the sister (age 59 years) demonstrated membrane-associated staining pattern comparable to control muscle (Figures 3B, C). Merosin protein levels as determined by Western blot were also comparable to controls (three age matched controls were used) in both subjects (Figure 3D). Merosin protein levels were found to be lower in the proband and sister compared to controls when normalized to actin and dystrophin but this difference was not statistically significant (Figure 3Ei, iii). There was a small increase in dystrophin protein levels when normalized to total protein in the proband and sister compared to controls but this difference was also not statistically significant (Figure 3Eii).

## Discussion

The phenotypic and genotypic spectrum of *LAMA2* muscular dystrophy is wide. Here we report a novel homozygous *LAMA2* variant in two siblings born to consanguineous parents. The *LAMA2* variant was not reported in several large reference population databases (7, 11, 12) and *in silico* analyses (13, 14) predicted the variant to have a deleterious impact on protein function (Table 1). The affected sister harbored the same variant in a homozygous state. Whilst genetic testing of the parents was not possible the LCSH on chromosomal microarray would support the novel variant occurring *in trans* due to known parental consanguinity. Collective clinical and genetic evidence supports classification of NM_000426.3:c.2906G>A;p.(Cys969Tyr) as likely pathogenic as per the ACMG-AMP criteria (15) (Table 1). A limitation of our report is the lack of *in vitro* or *in vivo* functional studies to assess for any damaging effect of this variant on the gene product. The variant identified in our study affects a highly conserved cysteine residue within the laminin-type epidermal growth factor (EGF)-like domain (16). Cysteines in this domain are critical for disulfide bond formation (16) and the variant is thus likely to affect the integrity of this rigid rod-like structure, which could destabilize the sarcolemma-extracellular matrix connections facilitated by the laminin-α2 protein (17). A study reporting 61 new LAMA2 variants and reviewing LAMA2 variants in the Leiden Open Variation Database (18) (https://www.LOVD.nl/LAMA2) found that 25% of deleterious missense variants affect cysteine residues within EGF-like repeats (17). A very rare missense variant [p.(Cys969Ser)] in the same amino acid position as our variant has been reported in the gnomAD database in the hetereozygous state without clinical disease (7).

The proband's clinical presentation was notable for profound weakness, severe joint contractures, extensive cerebral white matter changes, UMN findings, epilepsy, and dementia. Cerebral white matter changes are seen in nearly all patients with *LAMA2* muscular dystrophy (1, 2). In patients with adult onset LGMDR23, epilepsy is uncommon and cognition is usually intact despite the striking white matter abnormalities on brain MRI (1, 2). Mild deficits in executive function and mild cognitive impairment have been reported in a few older patients with adult onset LGMDR23 (19, 20). More significant cognitive impairment, in the form of intellectual disability, has been reported in LGMDR23 but only in younger patients with childhood onset disease (20, 21). The dementia seen in the proband and his sister could represent an extension of the LGMDR23 phenotype. However, as they were born to consanguineous parents it is possible that the dementia is due to the inheritance of another, albeit unrecognized, recessive pathogenic variant, especially given the LCSH seen on chromosomal microarray.

The finding of brisk reflexes and pathologic ankle clonus in the proband implies co-existent UMN pathology. This could be related to the extensive white matter changes, but UMN signs have not been described in other patients with *LAMA2* muscular dystrophy most of whom have similar white matter abnormalities on brain MRI.

The muscle biopsies from our subjects showed non-specific myopathic and dystrophic features consistent with the muscle biopsy findings in other LGMD23 cases (2, 4, 19, 20). However, most LGMD23 cases show at least partial merosin deficiency on immunohistochemistry and Western blot (2). Significant variability of merosin immunostaining in cases of LGMDR23 is well recognized and often depends on the antibodies used (22). However, the finding of normal merosin immunostaining, as seen in our subjects, is atypical. Normal merosin immunostaining is not unprecedented and has been described in two cases of genetically confirmed LGMDR23 using the same antibodies (MAb1922, Chemicon and NCL-merosin, Novocastra) (20, 23). Like our subjects, both cases had a late onset of symptoms with one case developing LGMD in their forties (20). In one of these cases, a subtle reduction of merosin immunostaining was only detected when the antibody directed against the N-terminal 300-kDa fragment of laminin-α2 (4H8, Alexis) was used (23). This differential immunostaining is consistent with the findings in other cases of LGMDR23 where staining with the N-terminal antibody often shows more abnormal results compared to staining with the antibody against the C-terminal 80-kDa fragment (MAb1922, Chemicon) (22). This could provide a possible explanation for the “normal” merosin immunostaining seen in our subjects as the N-terminal antibody was not used.

To our knowledge the findings of normal merosin Western blot in muscle despite clinical and genetic evidence of *LAMA2* muscular dystrophy has not been described. It is possible that a small change in merosin protein levels is not detected by our Western blot. Merosin protein levels were lower in the proband and his sister compared to controls when normalized to actin and dystrophin but these differences were not statistically significant (Figure 3Ei, iii). It is possible that the *LAMA2* variant [p.(Cys969Tyr)] could cause disease by conferring a toxic gain-of-function effect at the protein level, although this phenomenon would be extremely rare in the setting of a recessive disorder. Alternatively, the variant may reduce the activity of the laminin type EGF-like domain as postulated above but not significantly impact overall protein levels.

In the absence of published diagnostic criteria for *LAMA2* muscular dystrophy the finding of rare predicted to be pathogenic recessive variants in the *LAMA2* gene is currently considered to be the gold standard for establishing a diagnosis (1, 2). The presence of limb girdle muscle weakness, contractures, raised CK and cerebral white matter changes in the proband and his sister combined with likely pathogenic *LAMA2* variants are highly suggestive of LGMDR23. In particular, the finding of cerebral white matter changes in both subjects provides a compelling argument that the recessive *LAMA2* variants are the cause for their muscle weakness, as cerebral white matter changes appear to be unique to this sub-type of LGMD.

## Conclusion

This case report expands the genotypic and phenotypic spectrum of LGMDR23 through the identification of a novel homozygous *LAMA2* variant in a sibling pair with typical LGMD23 features including white matter signal changes–along with upper motor neuron findings and dementia. This finding highlights the utility of WES in aiding the diagnosis of rare neuromuscular disorders, particularly where the muscle biopsy results are inconclusive.

## Data availability statement

The datasets presented in this study can be found in online repositories. The names of the repository/repositories and accession number(s) can be found in the article/ Supplementary material.

## Ethics statement

The studies involving human participants were reviewed and approved by Human Research Ethics Committees of the Children's Hospital at Westmead, Australia (2019/ETH11736). The patients/participants provided their written informed consent to participate in this study.

## Author contributions

MK was responsible for conception, organization, and writing of manuscript. LW and SB were responsible for obtaining initial muscle and genetic data and assisted with editing of the manuscript. MY assisted preparation of the methods section on muscle immunohistochemistry and western blot and provided Figure 3 on muscle histopathology results and helped edit the manuscript. FG assisted with Table 1 on genetic variant, submission of genetic variant to ClinVar database, and helped edit the manuscript. SC was responsible for supervising collection, interpretation of muscle and genetic data, and assisted with editing of the manuscript. EO, TR, RH, and PM helped with editing of the manuscript. All authors contributed to the article and approved the submitted version.
